# Principles of safe day case appendicectomy and patient satisfaction rates: a systematic review

**DOI:** 10.1136/tsaco-2025-002054

**Published:** 2026-08-19

**Authors:** Arkeliana Tase, Alan Askari, Seiver Karim, Md Tanveer Adil

**Affiliations:** 1 Imperial College London, London, UK; 2 Luton and Dunstable Hospital NHS Foundation Trust, Luton, UK

**Keywords:** acute pain, Ambulatory Care, Appendicitis, appendectomy

## Abstract

**Background:**

Acute appendicitis is one of the most common surgical pathologies. While literature reports safe day-case appendicectomies in uncomplicated cases, hesitancy exists in discharging patients on the day of surgery. This study addresses the criteria for day-case surgery, patient selection, reported clinical risks versus benefits, complications, rates of readmission and patient satisfaction.

**Methods:**

A literature review was carried out in PubMed and Medline for day-case appendicectomy, patient safety, day case surgery and National Health Service. British Association of Day Surgery recommendations were reviewed in relation to appendicectomy as a day-case.

**Results:**

From the 146 identified papers, 19 fulfilled the inclusion criteria. Variability was noted in the definition of same-day discharge. Included patients had uncomplicated appendicitis, postoperative pain well managed by non-opioid analgesia, no clinical concerns and care at home. Age under 65 years, American Society of Anaesthesiologists (ASA) 1 or 2, absence of insulin-dependent diabetes and immunosuppressant therapy were also part of the inclusion criteria. No statistically significant difference was found in rates of reintervention and reoperation between admitted and discharged patients. Reasons for overnight admission were due to patient (age, comorbidities, etc) and system-related factors (theater availability and standardized pathways). Readmission occurred for wound infection, wound hematoma and minor bleeding. Patient satisfaction rates were high (59.4% to 91.2%).

**Conclusions:**

Day-case appendicectomies are safe and achievable with robust patient selection criteria, clear pathways and improvements in theater availability and education of staff and patients. Clear guidelines are required with standardized care pathways to safely achieve day-case discharge of patients after appendicectomy.

**WHAT IS ALREADY KNOWN ON THIS TOPIC**

- Guidance from the British Association of Day Surgery states that uncomplicated appendicectomy can be safely performed as a day case. This is, however, not widely practiced.

**WHAT THIS STUDY ADDS**

- This study shows that day-case appendicectomy can be safely performed with careful patient selection, clear protocols and systems in place and staff and patient education.

**HOW THIS STUDY MIGHT AFFECT RESEARCH, PRACTICE OR POLICY**

- The knowledge gained could be used to improve service efficiency while maintaining a high standard of care with better resource utilization.

## Introduction

 Acute appendicitis has a reported incidence of 7%–8%.^1 2^ It is one of the most common surgical conditions affecting children and adults.^3^ It most commonly occurs between the ages of 25 and 35 years old.^4^ According to Hospital Episode Statistics (HES) data, an average of 45 000–50 000 appendicectomies are performed in the UK every year.^5^

Day-case surgery has been an established pathway for a number of procedures such as laparoscopic cholecystectomy,^6^ incisional hernia repair^7^ and even major procedures such as Roux-en-Y gastric bypass.^8^ Enhanced recovery programs (ERAS) have been applied to elective surgery with improved patient outcomes. Modified ERAS protocols have also been studied to determine their adaptability to the emergency setting for the above procedures.^4 9^

Uncomplicated appendicitis has been defined in the literature as the absence of a hole in the appendix or fecalith in the abdomen or presence of an intra-abdominal abscess.^3 10^

Prior studies have reported that same-day discharge for uncomplicated appendicitis is safe and can reduce costs in both children and adults.^3 8 10–12^ However, hesitancy still exists in the application of same-day discharge for this group of patients.^8^

Challenges exist in establishing principles of safe same-day discharge in patients after appendicectomy and designing protocols, which are feasible and standardized to overcome these challenges and resistance from patient and clinical teams.

### Objectives

This study aimed to evaluate published literature on the criteria for day-case surgery, patient selection, reported clinical risks, benefits, complications and rates of readmission following day-case appendicectomy. Additionally, levels of achievable safe same-day discharge and patient satisfaction with the process were noted.

## Methods

### Information sources

A literature search was carried out in PubMed and Medline for day-case appendicectomy/appendectomy, patient safety and selection criteria. All papers published to date were included. Papers not in English and those only published as an abstract were excluded. Covidence Review tool was used to conduct the process of literature review and study selection.

### Eligibility criteria

All studies discussing criteria for day-case surgery, patient safety, risk benefits, complication rates and patient satisfaction were included.

Although we originally intended to only include studies published in the last 10 years to ensure up to date information, a further hand search identified six further studies published earlier than this time limit. After review, it was decided to expand the inclusion criteria to include these studies given their relevance to the topic.

Studies were excluded if they did not include a criteria/protocol for day-case surgery, presented a single case report, abstracts or did not discuss same discharge.

Studies including both adults and children were included as both were considered relevant to everyday practice despite their recognized differences to achieve a more round conclusion on the safety of day-case appendicectomy in all patient groups.

### Search strategy

Search criteria included MeSH terms for day-case appendicectomy/appendectomy, patient safety, day-case surgery and National Health Service. Additional publications were hand searched based on their relevance to the study aims. Data on national levels of appendicectomies carried out in the last 10 years were acquired from the HES database. Additionally, data published by the British Association of Day Surgery (BADS) were reviewed for levels of day-case surgery currently reported.

### Selection process

The studies were selected based on their relevance to the study objectives. The selected abstracts were reviewed by two of the authors independently (AT and AA), and decisions on inclusion/exclusion of studies were discussed, and an agreement was reached.

### Data collection process

Data collected from the studies were tabulated using Microsoft Excel to allow for analysis and presentation. Data were initially collected by one of the authors (AT), and a section of the papers was also reviewed and data collected by a second author (AA) to reduce bias in decision-making.

### Collected data items

Data were collected on the number of patients included in each study, age group, mode of surgery (laparoscopic vs open), length of stay, rates of successful same-day discharge, rates of complications, readmissions and reinterventions, rates of ED presentation and patient satisfaction with same-day discharge. The effect measures reported in each study are included in online supplemental table S1.

### Synthesis methods

Once the search was completed, the list of references was uploaded in Covidence software. Abstract review was carried out with the selection of studies being made based on the inclusion and exclusion criteria of the study. Those studies initially selected underwent full-text review. The final studies were selected based on the inclusion criteria. Data from individual studies were tabulated and presented in online supplemental table S1. Missing data were recorded as ‘not reported’ on online supplemental table S1.

### Risk of bias

Risk of bias was assessed by two independent reviewers using the Cochrane Risk of Bias 2 tool for the two randomized controlled trials (RCTs). All disagreements were resolved through discussion. The risk of bias for the cohort studies was assessed by using the ROBINS-I tool. Only studies with clear inclusion/exclusion criteria were included to reduce the baseline confounding bias.

Studies that did not have strict inclusion/exclusion criteria were excluded as considered to have a high risk of reporting bias.

### Ethics statement

Ethics approval not applicable.

## Results

### Identified studies

**Figure 1:**
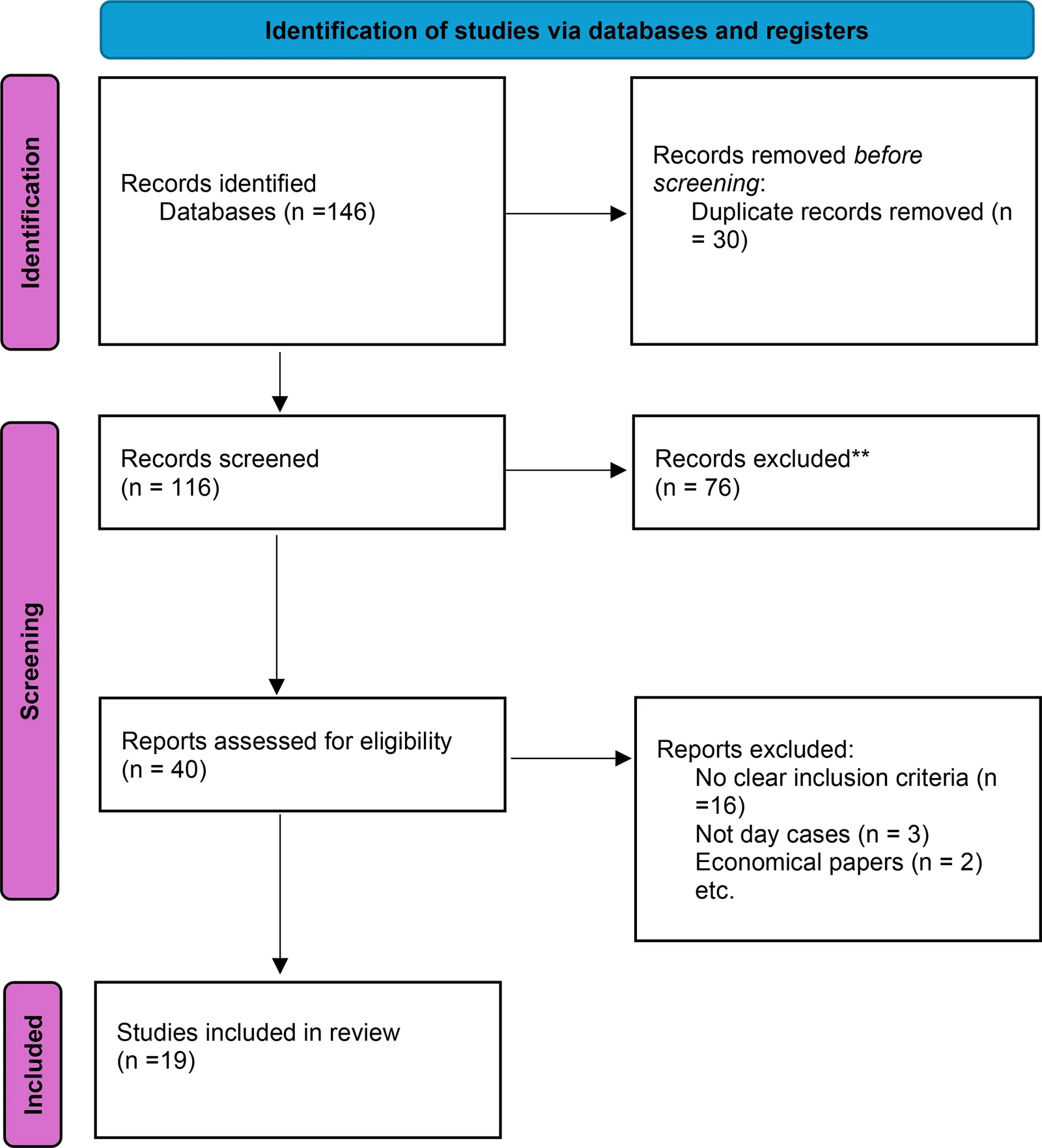
PRISMA flow diagram. PRISMA, Preferred Reporting Items for Systematic Reviews and Meta-Analyses.** Not fullfilling inclusion criteria.

The literature search generated 146 published papers. After removal of duplicates, 116 studies were abstract screened. 40 underwent a full-text review from which 19 fulfilled the study criteria (figure 1). The included studies discussed patient safety, feasibility, complication rates, rates of readmission and reintervention and patient satisfaction with the process. Nine of the studies solely included pediatric populations, 6 included both children and adults and the remaining 4 studies included only adult patients.

Appendicectomies were reported to be carried out by residents/fellows or general surgical trainees with supervision on five of the pediatric studies. One study included a large data review of patients operated on a large number of centers (both tertiary and regional/local units). The remainder of studies including pediatric patients did not specify the level of the surgeon carrying out the procedure.

The main difference noted between the adult and pediatric cases was the exclusion criteria, which was wider in the adult population. This difference was due to the fact that adult patients are more likely to have a number of comorbidities requiring longer postoperative monitoring. Additionally, some pediatric cases may be looked after in a tertiary center, which may impact the rates of day-case surgery compared with a non-specialist unit. The studies that included both adults and pediatric populations did not distinguish between the two on their outcomes.

### Definition of same-day discharge

Variability was found in the definition of same-day discharge between studies. Some authors calculate this from the date of surgery while others do so from the day of admission to the hospital. In the included studies, patients admitted late in the evening or night were operated on the next morning. The majority of authors argued that this affects the length of stay for non-surgical reasons. Due to these differences, it was not possible to compare the exact length of stay between studies without a large degree of bias.

### Rias of bias assessment

All studies were reviewed by two separate assessors (AT and AA). The same process was followed for the data collection section. Given the variability between studies and the reported outcomes, a strict inclusion criterion was followed to ensure that the reporting bias was low. Some studies were missing data on some of the outcome measures, and this was clearly noted on online supplemental table S1. Loss to follow-up was not recorded by any of the studies, and neither was the rate of negative appendicectomies. This is part of the study limitations.

### Study characteristics

Most of the studies (n=14) were cohort studies, two review studies, one case report, and two RCTs. From the randomized controlled trials, one of them^4^ was a single-center, unblinded study, which only included 48 patients in the treatment group and 49 in the control group. Three of the retrospective cohort studies^13–15^ included data from national databases; hence, they included a large number of patients. Only two of the studies included open appendicectomy,^14 16^ although they did not distinguish between open and laparoscopic procedures in terms of results. The remainder of the studies only included laparoscopic procedures. The inclusion and exclusion criteria were clearly defined and similar in all studies.

### Reported patient selection criteria

All studies define the inclusion criteria for uncomplicated appendicitis as patients whose pain is well managed by non-opioid analgesia, have no clinical concerns prior to discharge, and have a carer/relative to look after them in the first 24 hours after discharge. Other authors^16 17^ also required that patients are under the age of 65, live within 1 hour of the hospital, are ASA (American Society of Anaesthesiologists) 1 or 2 and likely to comply with the given advice.^16^

Exclusion criteria included pregnant patients, insulin-dependent diabetic patients and those on immunosuppressive medications.^16 17^ Table 1 demonstrates the summary of inclusion and exclusion criteria reported in the studies.

**Table 1:** Patient selection criteria

Inclusion	Exclusion
Uncomplicated appendicitis	Age>65 years old
Stable observations postoperatively	Perforated, gangrenous appendix, peritonitis
Pain well managed with non-opioid analgesia	Conversion to open procedure
Tolerating oral intake	Unstable observations, fever, tachycardia
Responsible adult available to stay with patient overnight	Inability to tolerate oral intake
Lives within 30 min of hospital	Patients requiring opioid analgesia postoperatively
Has received educational leaflet and information required	Inability to pass urine postoperatively
No drain inserted	Pregnant patient
	Insulin-dependent diabetes
	Patient on immunosuppressive medication or other medication that requires monitoring

### Reported rates of uncomplicated appendicitis

Rates of reported uncomplicated appendicitis were high and varied between 51% and 85.7% (table 2) Given the variability in patient size between studies, the n-weighted mean was calculated and found to be 69.68% for the laparoscopic surgery. In this case, the ‘weight’ is the number of patients included in each study.

**Table 2:** Reported rates of uncomplicated appendicitis by study

Author	Total number of patients	Reported rates of uncomplicated appendicitis
Fuad Alkhoury *et al*^19^	251	82.4% (Lap)
Benedict *et al*^20^	569	80% (Lap)
Dubois *et al*^17^	109	63% (Lap)
Gilliam *et al*^23^	104	80.3% (Lap)
Halter *et al*^21^	304	78% (Lap)
Oyetunji *et al*^15^	40 263	71.3% (Lap)
	15 813	54.5% (open)
N- weighted mean		69.68%

Lap, laparoscopic surgery.

### Rates of emergency department presentation and readmission

The rates of readmission and emergency department presentation were reported to be low. They ranged from 0% to 20% (IQR 0.85%–2.45%) with no statistically significant differences between overnight admissions and day-case appendicectomy patients (online supplemental table S1). The single study^3^ reporting a 20% rate of ED presentation for SDD also reported a 16% rate of ED presentation for the admitted patients with no statistical difference between the two groups (p=0.61).

The rates of reintervention (surgery) were reported to be very low (0%–2.4%). Two studies^13 18^ reported the use of interventional radiology drains for treatment of intraabdominal abscesses. No statistical difference was reported between the two groups except for one study^13^ that reported statistically significant higher rates of reoperation in the admission group compared with the same-day discharge one (17.6% vs 0% p=0.024)

Laparoscopic appendicectomy was found to be 50% more likely to lead to a same-day discharge compared with open surgery.^15^ The most common reasons for readmission were reported to be wound infection, wound hematoma, minor bleeding and intraabdominal collection.

### Reasons for overnight admission postoperatively

The main factors affecting same-day discharge were similar between studies. These were grouped into patient-related and system-related factors.

The main *patient-related factor* was the time of presentation to hospital. Patients admitted after 18:00 and overnight were often operated on the next morning unless clinically unwell, which in turn affected the time of discharge. Patients undergoing appendicectomy earlier in the day were more likely to be discharged on the same day as the operation. Alkhoury *et al*^19^ reported that 77.8% of patients admitted overnight did so solely due to the surgery finishing late in the evening. Only 11% were admitted for medical reasons (nausea, vomiting, pain), and 11% were admitted as the families refused to leave the hospital. Benedict *et al*^20^ reported that 16% were admitted due to pre-existing medical comorbidities. Dubois *et al*^17^ also found that pre-existing comorbidities were a significant reason for admission (37% vs 15%). This group of patients was also found to be more likely to experience postoperative complications (14% vs 3%).

Female patients were reported to be more likely to be admitted compared with their male counterparts due to gynecological pathologies being the cause of the abdominal pain and hence persisting postoperatively.^14^ As a result, women were found to be 47% less likely to be treated as day cases (p=0.005) due to the primary etiology being non-appendicitis.^14^

*System-related factors* such as theater availability, standardization of care pathways and better education of staff, patients and relatives also played an essential part in achieving successful day-case discharge. By improving these factors, Juviler *et al*^3^ increased the rates of same-day discharge from 32% to 75%. This improvement occurred with no statistically significant difference between readmission and emergency presentation rates.^3^

Box 1 demonstrates a summary of the reported reasons for hospital admission.

Box 1 **Reported reasons affecting same-day discharge**

**Patient-related factors.**

Time of admission–between 18:00 and 6:00.

Time of operation—between 12:00 and overnight.

Female patients.

Pain, nausea, vomiting, not tolerating oral intake.

Social factors—inadequate living conditions.

**System-related factors.**

Theater availability and prioritization of cases in the morning.

Standardized pathways for day-case appendicectomy.

Staff education.

Postoperative day-case discharge unit/ambulatory unit.

### Reported patient satisfaction

Six studies reviewed reported measures of patient/parent satisfaction with day-case discharge. Rates of satisfaction on discharge ranged from 59% to 91.2% (table 3). Two studies^19 21^ repeated the measures of satisfaction during follow-up appointments. In retrospect, patients reported higher rates of satisfaction with same-day discharge (95% from 87% and 78% from 59.4%, respectively) when asked at the follow-up appointment. The main reasons affecting levels of satisfaction were anxiety about being discharged home soon after the surgery, fear and anxiety regarding pain management and worry about seeking help in case of concerns. Pain was reported to be well managed with non-opioid analgesia.^10^

**Table 3:** Reported rates of patient satisfaction

Author	Date	Satisfaction rates
Nelimar Cruz-Centeno *et al*^10^	2023	89.9%
Fuad Alkhoury *et al*^19^	2012	87%
		(95% on retrospect)
Cosse *et al*^16^	2014	91.2%
Lopez *et al*^4^	2022	59.4% happy to go
		28.1% nervous but OK
Gee *et al*^12^	2021	78.1%
Halter *et al*^21^	2016	59% happy to go
		28% nervous but OK
		80%—in retrospect overall happy

## Discussion

Published literature demonstrates that day-case discharge after laparoscopic appendicectomy is safe and feasible.^22^ It is associated with low rates of complications, readmissions and reinterventions when patient selection is carried out appropriately and system-related factors such as theater availability and standardized pathways are addressed. Generally, the rates of uncomplicated appendicitis are high with rates of perforated or gangrenous appendicitis occurring in 15%–20% of cases.^23^ The results of this study can be applied to many units that may not have a specified pediatric surgery service. This is because surgeries in the pediatric unit were reported to be carried out by surgeons of different training levels (fellow/resident/general surgery trainees) with no differences in their protocols for patient management, including their inclusion criteria. Furthermore, the reported levels of postoperative complications, readmissions and returns to theater were similar between studies.

A number of different scoring systems have been developed to assess the severity of acute appendicitis. They include the Appendicitis Inflammatory Response, Alvarado score, Ripasa score among many others.^24^ Comparison of the different scoring systems has shown variable sensitivity and specificity between them. The Alvarado score, for example, is reported to have a sensitivity of 83% and specificity of 47%.^6^ These scoring systems are not regularly used in clinical practice. None of the studies reviewed included a scoring system as a method of patient selection. Their decision-making was based on patient observations, clinical and operative findings and specific patient-related factors such as existing comorbidities, insulin-dependent diabetes and age over 65 years among others as discussed above. Hence, it can be argued that the existing scoring systems, while helpful to some extent are not sufficient in the diagnosis of uncomplicated appendicitis. Currently, many patients (mainly adults) undergo CT scanning as part of the initial investigations. This can assist in predicting complicated versus uncomplicated appendicitis; however, the best decision appears to be made based on perioperative findings.

In 2019, BADS^25^ introduced a table of day-surgery procedures that are suitable in relation to emergency surgery. For general surgery, these include incision and drainage of abscess, laparoscopic cholecystectomy, laparoscopic appendicectomy and temporal artery biopsy. They advise that patients are operated on emergency theaters and follow the day surgery pathway postoperatively for those patients fulfilling the selection criteria. These procedures can be successfully performed as day cases provided a good pathway is in place. This includes a surgical assessment unit for shared decision-making.^26^ Currently, 200 procedures are listed as suitable for day-case surgery.^26^ Emphasis is placed on the evaluation of existing inpatient procedures and pathways with decisions made on aspects that require modification to allow day-case surgery to take place.^26^

Emphasis is placed on the importance and role of the Multi-Disciplinary Team in achieving safe-day surgery and the success of the set pathway. Team training and education with clear protocols are advised as an essential step in successfully achieving required goals. Admission to a day surgery unit is reported to be more likely to achieve day-case discharge compared with admission in an inpatient unit (2.4% vs 14%).^26^

According to BADS, the decision for day surgery is influenced by (1) the patient’s ability to tolerate oral intake, (2) pain well managed by oral analgesia and (3) the procedure should have a low risk of postoperative complications. They need to be ASA I or II with no significant comorbidities, neonates or premature infants. Additionally, patients should have a place of residence, live a reasonable distance from the hospital and have someone available to care for them in the first 24 hours postoperatively.^26^ These requirements reflect those found in literature reported in this study.

Limitations to day case discharge exist for certain groups of population such as those above the age of 65 years old. This relates to pre-existing comorbidities that require close monitoring postoperatively. It is, however, important to recognize that long hospital stays without a good clinical cause can have a negative effect in loss of muscle function (5% loss of muscle function for every day in hospital) in those aged over 65 years.^27^ Similarly, patients with a high BMI (body mass index) greatly benefit from day surgery, which has been shown to reduce risks of venous thromboembolism and hospital-acquired infections.^28^

Setting the right expectations with patients/parents prior to surgery improves rates of successful same-day discharge in children and adults.^3^ Authors have reported variability in patient management between different hospitals and clinical teams. While this is inevitable, standardizing care pathways would increase the rates of same-day discharge.^18^

### Study limitations

The study limitations relate to a number of points. First, most of the published literature consists of cohort studies (both retrospective and prospective). While most of the studies state differences in outcomes between day case and non-day case patients, the vast majority compare the numbers with historical outcomes often not stating the number and characteristics of the historical patients. The number of randomized controlled trials is limited with only two of the search results showing the presence of RCT with limited outcomes. Second, the definition of same-day discharge varies between studies. Some studies define this as discharge on the same day as the admission while others define it as discharge on the day of the surgery (which may not be the same as the admission date). Additionally, no standardized method of reporting the published studies was noted. For example, some studies report ORs, others report percentages of outcomes and the remaining ones report actual numbers. This creates difficulties in placing the data together for further statistical analysis. The studies that include both pediatric and adult patient populations do not distinguish between the results of these two groups, which makes it difficult to carry out any further analysis based on age groups.

## Conclusions

Safety and feasibility of day case appendicectomy for uncomplicated appendicitis in both children and adults is established by published literature. By applying safe selection criteria, improved pathways and organizational setup and patient and staff education, day-case surgery for emergency appendicectomy can be carried out safely with benefits to patients and healthcare trusts.

Clear guidelines are required to aid members of the surgical team and patients in this process. Reaching a consensus and standardizing care pathways can aid in achieving successful day-case discharge without compromising patient care.

## Supplementary material

10.1136/tsaco-2025-002054 online supplemental table 1

## Data Availability

All data used in this review study are publicly available
