# Supplementary material for: Principles of safe day case appendicectomy and patient satisfaction rates: a systematic review
Source: Trauma Surg Acute Care Open. 2026 Aug 19;11(3):e002054. doi: 10.1136/tsaco-2025-002054 (PMC13504945; doi:10.1136/tsaco-2025-002054)
Supplement: Supplementary file 1 [file tsaco-11-3-s001.docx]

*Table S1: Summary of data from the included studies*

| **Author** | **Type of study** | **No of patients** | **Median age** *(years)* | **Open/ Laparoscopic** | **LOS/** *hours* | **Children/**  **Adults** | **Successful**  **SDD** | **Complications (SDD vs admitted)** | **Readmission Rates** | **ED**  **presentation** | **Re-intervention rates** |
| --- | --- | --- | --- | --- | --- | --- | --- | --- | --- | --- | --- |
| Rates Nelimar Cruz-Centeno et al ^1^ 2023 | Cohort | 255 | 12 | Laparoscopic | 3.8 | Paediatric | 100% | NR | 0% | 1.55% | 0% |
| Fuad Alkhoury et al^2^ 2012 | Cohort | 158 | 12 | Laparoscopic | 4.8 | Paediatric | 80% | 2% vs 3%  Wound infection | 0% | 2% vs 3% | 0% |
| Fuad Alkhoury et al ^3^ 2012 | Cohort | 207 | 12 | Laparoscopic | 5 | Paediatric | 73.8% | 3.7% vs 6.7%  Wound infection | 2.5% vs 2.2% | 7.4% vs 4.4% | 0% |
| Benedict A et al ^4^ 2018 | Case control | 569 | 11 | Laparoscopic | 4 | Paediatric | 87% | 1.6% vs 4% | 2% vs 4% | 0% | 0% |
| Buss et al^5^ 2022 | Cohort | 972 | 12.1 | Laparoscopic | 7 | Paediatric | 36.9% | NR | 2.2% vs 5% | 3.4% vs 6.9% | 0% |
|  |  | 1 |  | Open |  |  |  |  |  |  |  |
| Cosse et al^7^ 2014 | Review | 1063 | NR | Laparoscopic | 12 | Adults | 83.42% | 3.16% | 2.01% | 3.16% | 0-2.5% |
|  |  | 318 |  | Open |  |  |  | 0% |  |  |  |
| Devin C L et al^8^ 2020 | Cohort | 268 | 11.3 | Laparoscopic | 2.6 vs 10.6 | Paediatric & adult | 46.7% | 1.6% vs 0.4% (intra-abdominal infection)  2.6% vs 1.1%  Wound infection | 3.3% vs 1.5% p=0.07 | 5.2%  Vs 5.5 % | 2.41 vs 0.71 (odds ratio)  Percutaneous drain insertion |
| Dubois et al^9^ 2010 | Cohort | 161 | 29 | Laparoscopic | 4.7 | Adults | 66% | 10.6% vs 13.5%  *P=0.49* | NR | 11% vs 14%  P=0.39 | NR |
| Lopez J E et al^10^ 2022 | RCT (single centre , unblinded) | 48 SDD vs 49 control | 32.2 | Laparoscopic | 30 | Adults | 87.7% | 1.75% vs 11% | 0% | NR | 0% |
| Farach S M et al^11^ 2014 | Cohort | 206 | 11.5 | Laparoscopic | 3.1 | Paediatric & adults | 90% | 2.7% vs 11% | NR | NR | NR |
| Gee K M et al^12^ 2021 | Cohort | 849 | NR | Laparoscopic | 8.1 vs 21.5 | Paediatric | 40% | 2% vs 6%  *P=0.219* | 0.5% vs 1%  *P=0.21* | 22pts vs 27pts *p=0.98* | NR |
| Gilliam A D et al^13^ 2008 | Cohort | 104 pts | 22 | Laparoscopic | 25 | Paediatric & adult | 66.64% | 0% | 2.4%  Pelvic abscess | 0% | 2.4% |
| Grigorian A et al^14^ 2019 | Retrospective cohort | 16,931 | NR | Laparoscopic | 33 | Adults | 23.6% | 1% vs 1.5% | 1.8% vs 2.3%  *P=0.051* | NR | 56% vs 40.7%  *P=0.171*  *IR intervention*  0% vs 17.6%  *Re-operation* |
| Gurien L A et al^14^ 2017 | Cohort | 171 | 11.2 | Laparoscopic | 3.1 | Paediatric | 92% | 1 vs 0 | 0% | 13 vs 0  P=0.98 | NR |
| Halter J M et al^15^ 2016 | Cohort | 236  (121 SDD) | 11.1 | Laparoscopic | 11.8 | Paediatric | 51% | 0.8% vs 2.6%  P=0.3 | 0.8% vs 2.6%  P=0.35 | 6.7% vs 2.6%  *P=0.174* | NR |
| Juviler P et al 2023 | Cohort | 264 | 11.4 | Laparoscopic | 5.7 | Paediatric | 75% | NR | 7% vs 3%  *P=0.212* | 20% vs 16%  *P=0.491* | NR |
| Oyetunji T A et al 2015 | Cohort | 40263 | 14 | Laparoscopic | 24 | Paediatric and young adults (<20yrs) | 71.3% | NR | NR | NR | NR |
|  |  | 15814 |  | Open |  |  | 54.6% |  |  |  |  |
| Duran et al 2024 | RCT | 149 SDD vs 151 control | >14 | Laparoscopic | 24 | Paediatric & Adult | 85.9% | 4.7% vs 5.3% | 4.7% vs 1.32% | NR | 0% vs 1.3% |
| Kashyap M V et al 2021 | Review | 511 SDD, 930 Overnight | 11.5 | Laparoscopic | NR | Paediatric | 35% | 1% vs 3% | 0.9% vs 1%  *P=0.82* | 2.6% vs 2.1%  *P=0.61* | 0.6% vs 1%  *P<0.01* |

*SDD – Same Day Discharge

*all studies reported including only cases of uncomplicated appendicitis as their selection criteria

*ED – emergency department

**NR – not reported
